# P-466. Clinical and Microbiological Characteristics of Secondary Hemophagocytic Lymphohistiocytosis in People Living with HIV/AIDS

**DOI:** 10.1093/ofid/ofae631.665

**Published:** 2025-01-29

**Authors:** Xavier A Flores-Andrade, Amy B Peralta-Prado, Alvaro López-Iñiguez, Gonzalo Salgado-Montes de Oca, Jesús Delgado-de La Mora, Andrea Cárdenas-Ortega, Angel G Vargas-Ruiz, Ethel Cesarman, Santiago Ávila-Ríos

**Affiliations:** National Institute of Respiratory Diseases "Ismael Cosio Villegas" (INER), Mexico City, Distrito Federal, Mexico; National Institute of Respiratory Diseases "Ismael Cosio Villegas" (INER), Mexico City, Distrito Federal, Mexico; Instituto Nacional de Ciencias Médicas y Nutrición Salvador Zubirán., Mexico city, Distrito Federal, Mexico; National Institute of Respiratory Diseases "Ismael Cosio Villegas" (INER), Mexico City, Distrito Federal, Mexico; Weill-Cornell Medicine, New York City, New York; National Institute of Respiratory Diseases "Ismael Cosio Villegas" (INER), Mexico City, Distrito Federal, Mexico; National Institute of Respiratory Diseases "Ismael Cosio Villegas" (INER), Mexico City, Distrito Federal, Mexico; Weill-Cornell Medicine, New York City, New York; National Institute of Respiratory Diseases "Ismael Cosio Villegas" (INER), Mexico City, Distrito Federal, Mexico

## Abstract

**Background:**

Hemophagocytic lymphohistiocytosis (HLH) or commonly known as hemophagocytic syndrome, is a rare and serious disease triggered by a myriad of stimuli. HLH is characterized by an uncontrolled cytokine production, persistent lymphocyte and macrophage activation which results in hemophagocytosis. Since people living with human immunodeficiency virus (PLHIV) have an increased risk of coinfections, HLH is gaining great relevance in the clinical field as it worsens patient's prognosis.

**Figure 1.**
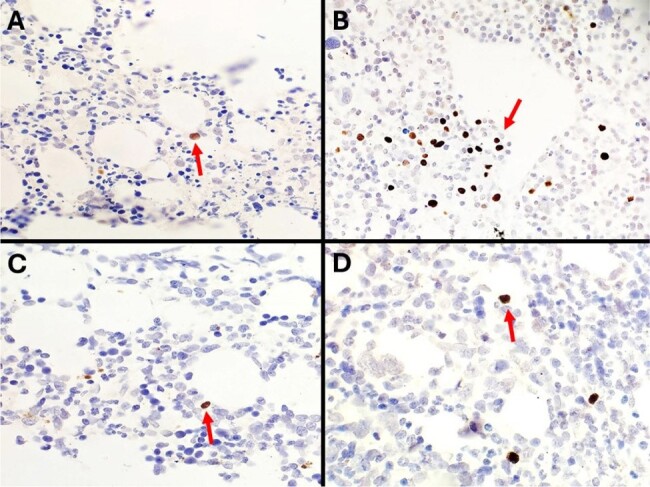
Epstein-Barr virus-encoded small RNAs (EBER) in situ hybridization in bone marrow biopsies. A-D Microscopic examination (EBER) of bone marrow biopsies. EBER was performed to identify the presence of Epstein-Barr Virus in bone marrow specimens. EBER expression was identified in medium and large lymphoid cells (red arrows) in all samples in different proportion: <1% (A and C, 10x), ∼30% (B, 10x), and ∼3% (D, 20x). EBV could be a significant trigger for HLH in conjunction with any other infectious or neoplastic triggers that the patient may present.

**Methods:**

We conducted a descriptive retrospective study to elucidate the main characteristics of secondary HLH in PLHIV. The analysis included 16 participants with more than 90% probability for HLH by HSCORE, in two reference centers in Mexico City from January 2018 to December 2023. Clinical and microbiological data were collected from clinical records, as well as bone diagnostic biopsies to perform Epstein–Barr virus–encoded small RNAs (EBER) in situ hybridization and to confirm infectious or neoplastic triggers.

**Results:**

From a total of 16 participants, 2 (13%) were cisgender women, 3 (19%) were transgender women, and 11 (69%) were cisgender men. A total of 6 (38%) participants presented immune reconstitution inflammatory syndrome and 6 (38%) died. Erythrophagocytosis was confirmed in 6 (38%) bone biopsies. Fungal diseases, such as *Histoplasma capsulatum* (n = 6 [38%]) and *Cryptococcus spp*. (n = 2 [13%]), were the most common trigger for HLH, followed by viral diseases, including influenza virus (n = 2 [13%]), cytomegalovirus (n = 2 [13%]), and other viruses such as hepatitis B virus, adenovirus, rhinovirus, SARS-CoV-2, and Mpox. Mycobacterial disease was observed in 3 (19%) individuals. A total of 4 (25%) participants presented with neoplastic triggers, 3 (19%) of which had Hodgkin's lymphoma, and 1 (6%) non-Hodgkin lymphoma. EBER was performed in 4 bone marrow biopsies from which 4 were positive (Figure attached).

**Conclusion:**

Since HLH can progress to a devastating scenario in conjunction with other severe diseases that put the patient's life at risk, it is important to maintain surveillance of HLH development, especially in people presenting hematological malignancies and fungal and viral diseases, particularly EBV disease which is a common coinfection among PLHIV.

**Disclosures:**

**Andrea Cárdenas-Ortega, N/A, MD**, Exeltis: Speaking and educational

